# The Effect of Whitening Agents (Whitening Rinse and Carbamide Peroxide) on Stained Flowable and Packable Composite Aligner Attachments

**DOI:** 10.1007/s00784-025-06298-1

**Published:** 2025-04-04

**Authors:** Ezgi Atik, Ülkü Tuğba Kalyoncuoğlu

**Affiliations:** 1 Department of Orthodontics, Faculty of Dentistry, Hacettepe University, Ankara, 06100, Turkey; 2 Department of Prosthodontics, Gülhane Faculty of Dentistry, University of Health Science, Keçiören, Ankara 06018 Turkey

**Keywords:** Bleaching, Color, Whiteness index, Composite attachment

## Abstract

**Background:**

This study aimed to verify the effects of whitening agents on the color stability of aligner attachments made from flowable and packable composite resins after staining with coffee and wine.

**Methods:**

Two composite groups were used for attachment preparation on epoxy resin master models: G-ænial Universal Injectable flowable (Group 1, N = 42) and G-ænial Posterior packable (Group 2, n = 42). Initial staining of samples was performed with coffee and red wine respectively, with a total period of 6 days representing 6 months of consumption. After staining, each main group was randomly divided into 3 groups related to distilled water as the control (Distilled Water (DW), n = 14) and whitening agents (Whitening Oral Rinse (WR), n = 14) (Carbamide Peroxide 22% (CP), n = 14) for a 14-day test period. Before staining (T0), after staining (T1), and after whitening (T2), color measurements were made with Vita Easy Shade V. *ΔE*_*00*_ formula was used to determine color differences between T0-T1 (*ΔE*_*001*_*)*, T1-T2 (*ΔE*_*002*_*)* and T0-T2 (*ΔE*_*003*_*)* color measurement periods. Also, attachments’ whiteness change before and after whitening procedures was calculated using the Whiteness Index for Dentistry (WI_D_) formula considering as ΔWI_D1_ = WI_D_(T2) − WI_D_(T0) and ΔWI_D2_ = WI_D_(T2) − WI_D_(T1).

**Results:**

After immersion in staining solutions, ΔE_001_ values of Group 2 were significantly higher than of values of Group 1 (p < 0.001). In Group 1, ΔWI_D2_ values in the WR and CP groups were found to be significantly higher than measurements in the DW group (p = 0.049 and p = 0.001). In Group 2, the value of ΔE_002_ for subgroup CP was significantly higher than that for DW group (p = 0.023). Also, the ΔE_003_ measurement of the WR group was higher than the measurement of the CP group (p < 0.001). In Group 2, the ΔWI_D1_ measurement of the CP group was lower than the measurements of the DW (p < 0.001) and WR (p = 0.014) groups. According to ΔWI_D2_ measurement in Group 2, CP measurements were higher than DW (p < 0.001) and WR (p = 0.024) measurements. Statistically significant differences were determined between the composite types for DW, WR, CP whitening types in terms of ΔE_003_ and ΔWI_D1_ measurements and between the composites for CP whitening type in terms of ΔWI_D2_ measurements (p < 0.05). For all significant differences, measurements for Group 2 were found to be higher than for Group 1.

**Conclusion:**

The color change of packable composite was more pronounced than that of the flowable composite after staining. Between whitening and staining stages, especially in packable composite group, carbamide peroxide whitening agent significantly effected the color and whiteness of the attachments compared to control distilled water group.

## Introduction

Clear aligner treatment modality has gained popularity in recent years because of the quest for improved aesthetics. During treatment with clear aligners, attachments are mostly attached to the surfaces of the teeth to enhance the efficiency and predictability of tooth movements [1]. Resin-based dental composites are used to realize the attachments positioned in the virtual planning in the mouth, and it is very important to choose the right resin material in terms of aesthetic and mechanical necessities [2].

In clinical practice, composite resins of different viscosities with different filler amounts continue to be used in attachment production according to the clinician’s preference. Both flowable and packable resin composites may be preferred for creating aligner attachments with varying features regarding filler content, volume, and monomer composition [3]. Flowable resin composites exhibit lower viscosity due to the same small particle sizes combined with a reduced filler volume and increased resin content. [4]. The injectable nature of flowable composites offers enhanced handling properties and, enables the resin to be easily placed in the attachment template [4]. These features save time during the bonding of attachments and facilitate isolation during placement protocol. On the other hand, packable composites are composed of small particle sizes with a high filler volume and high viscosity, resulting in a better mechanical performance and greater resistance to deformations [5].

Because of the greatly enhanced knowledge of the patients about dental aesthetics, the demand for aesthetic tooth-colored restorations has increased [6]. Resin composite materials are subjected to different kinds of stains in the oral cavity [7, 8]. The color change of the resin-based composites can be affected by intrinsic and extrinsic factors, mainly incomplete polymerization, water absorption, chemical reactions, oral hygiene status, surface roughness of materials, finishing and polishing procedures, and dietary habits [7, 9–11]. Internal discoloration of the resins mainly arises from the chemical and physical reactions in the innermost layers of the resin, while external discolorations from different informants such as beverages, food, smoking, and application of mouthwashes [12, 13]. The most common beverages that cause different degrees of coloration on the surfaces of resin materials have been reported as Coke, red wine, fruit juices, coffee, and black tea in the literature [14–16].

The characteristics of the resin matrix content and inorganic filler particles significantly influence the smoothness and stain resistance of composite materials [17]. Since aligner attachments are not polished to avoid affecting tooth movement, their surface roughness may lead to additional discoloration of the attachments [18, 19].

Mouthwashes are widely used among patients as a popular over-the-counter tooth whitening measure due to their ease of use, low cost, and availability [20, 21]. They mainly involve hydrogen peroxide as the active ingredient [22]. These products do not require a prescription and are usually used daily over a period of 2 weeks. The other option is the home bleaching technique in which trays are used by patients at home, supervised by the dentist, by using 10 to 22% carbamide peroxide concentrations, which is considered as a safe cosmetic dental procedure [23].

During the bleaching process, reactive oxygen species from the bleaching agents may also interact with existing restorations in the mouth, which explains why several studies have investigated the effects of bleaching agents on the optical properties of restorative materials [10, 24–29]. Also, whitening oral rinses may lead to an increase in the surface roughness parameters of composite resins [30] and therefore leading to possible color changes on the surface of both tooth and adhesive restorations [31, 32].

There are limited studies in the literature regarding color changes of aligner attachments made of different composite resins after exposure to different staining solutions [2, 33–35]. Along with the increasing demand for aligner treatment, whitening of teeth is in greater demand frequently during or after aligner treatment [36]. However, to the best of our knowledge, there is no information regarding the influence of at-home bleaching agents and whitening oral rinse mouthwashes on the color stability of aligner attachments derived from different composites having different properties and viscosities. GC introduced G-aenial Universal Flo (GC Corporation, Tokyo, Japan*)* is a new generation of flowable universal composite resins with improved physical and mechanical properties, which offer easy handling and good viscosity. Homogeneously dispersed and extremely fine silane-treated filler particles in this resin [37] also make it possible to increase the gloss of unpolished surfaces over time due to their self-polishing property [38]. G-aenial posterior composite is a micro-filled hybrid composite combining two types of prepolymerized (organic) resin fillers and inorganic fillers. The prepolymerized fillers both reduce the volumetric shrinkage without reducing mechanical properties and increase the overall filler loading without increasing its volume [39, 40].

The null hypotheses of the present in-vitro study were defined as there would be no difference between carbamide-peroxide agents and whitening mouthwashes in terms of color changes on the aligner attachments derived from either G-aenial Universal Flo flowable or G-aenial posterior packable composite.

## Materials and methods

### Sample preparation

One master model, made of epoxy resin, printed before with a 3D-stereolithography printer (Formlabs Inc., Somerville, MA, USA) was used to create realistic upper arches to reproduce the realized aligner attachment placement procedure. Since epoxy resin models were used for the study, ethical approval was not required for this type of research. To duplicate the epoxy resin model, an intraoral Trios 3 (3Shape, Copenhagen, Denmark) scanner was used for the extraoral model scanning. Through this process, six models were created to be used in different subgroups. The manufacturer of the aligners (Orthero, Istanbul, Turkey) fabricated six templates to support the attachment realization, respectively, taking into account the STL image of the epoxy resin master model. The horizontal attachments with dimensions of 5 × 2x1.25 mm were placed on the middle third of each tooth using Orthero software, and appropriate attachment templates were produced in a virtual environment.

Sample size calculation was determined using G*Power software (G*Power v.3.0.10; Franz Faul, Universitat Kiel, Germany). Based on a previous study [41] and considering the statistically significant difference in *ΔE2*_*00*_ between two whitening agent types (for Opalescence Go whitening: 3.08 ± 0.80 and Whitening mouth rinse: 2.08 ± 0.68), the power analysis showed that with a presumed effect size of 1.35, with a significance level at 0.05 and 90% power, minimum 13 specimens for each subgroup were required for conducting this study.

On three epoxy resin master models (Group 1, N = 42 teeth), G-ænial Universal Flo (GC Corporation, Tokyo, Japan) flowable composite resin was used for bonding the attachments on the master models. In the other three epoxy resin master models (Group 2, N = 42 teeth), G-ænial posterior packable composite resin (GC Corporation, Tokyo, Japan) was used for bonding the attachments. These composites were chosen because of their preferences in the routine aligner treatment protocols and their different viscosities. A2 color was chosen for two types of composites. The composite attachments were cured for 3 s using a VALO Ortho LED (Ultradent Products, South Jordan, Utah) with 3200 mW/cm^2^. Attachment bonding was performed on each model, including all teeth from the right second molar tooth to the left second molar tooth, by the same practitioner (E.A.). A sample of 42 teeth in total was created on 3 master models in each two main groups (Group 1 and Group 2). All prepared samples were stored in distilled water at 37 °C for 24 h [42] to ensure completion of polymerization.

### Staining process

All the samples' baseline surface color (T0) values (N = 84) were measured before the staining process. Information about the whitening agents, composite resins, and their compositions is given in Table 1. Coffee (Nescafé® Original, Nestlé, Vevey, Vaud, Switzerland), prepared with 30 gr of instant coffee powder per 2.5 L of boiled distilled water, and red wine (Fiona, X) were used as coloring solutions. The specimens were placed in lid-fitted plastic boxes, fully immersed in the solutions corresponding to each group. The dimensions of these boxes were consistent across all samples. The immersion solutions were renewed daily, and the specimens were maintained in the incubator at 37** ± **1 °C for three days with each coloring solution, totaling a duration of 6 days. Six days of immersion were preferred to stimulate six months of consumption of each beverage, according to the study by Ertas et al. [43] After immersion in solutions, all specimens were rinsed with deionized distilled water and dried with air and absorbent paper. Color measurements (T1) were repeated after the staining process.

**Table 1:** Material details used in the study

Materials	Manufacturer	Formulations or ingredients	Application methods
Listerine Advanced White	Johnson&Johnson S.P.A, Pomezia, Italy	Aqua, alcohol, sorbitol, tetrapotassium pyrophosphate, pentasodium triphosphate, citric acid, poloxamer 407, sodium benzoate, eucalyptol, thymol, sodium saccharin, sodium fluoride, menthol, sucralose, tetrasodium pyrophosphate, propylene glycol, sucralose, aroma, disodium phosphate	Immersion for twice 1 min, each day
Pola night	SDI Limited, Bayswater, Victoria, Australia	Carbamide peroxide (22%); chemical additives (< 40%); glycerol (30%); water (20%); and flavoring (0.1%)	45 min each day
G-ænial Universal Flo (nano-hybrid flowable) (A2 shade)	GC Corporation. Tokyo, Japan	Bis-EMA, dimethacrylate co-monomers, UDMA, silica, barium glass, (filler content: 69 wt%/50 vol%)	According to the manufacturer instructions
G-ænial Posterior (micro-filled resin hybrid composite restorative) (P-A2 shade)	GC Corporation. Tokyo, Japan	UDMA, dimethacrylate co-monomers (Bis-GMAfree), Fluoroaluminosilicate glass, fumed silica, pre-polymerized fillers (silica, strontium and lanthanoid fluoride), (filler content: 77 wt%, 65 vol%)	According to the manufacturer instructions

### Application of whitening agents

In each composite group, the samples were randomly divided into three subgroups (n = 14) based on the whitening agents and storage conditions used as follows:

Group 1-DW (n = 14) and Group 2-DW (n = 14): All specimens in these groups were kept in distilled water at 37 °C in a dark environment for the entire study period of 2 weeks (the same duration as the whitening application) to act as control groups. These groups were created in order to evaluate intrinsic color changes within the restorative materials.

Group 1-WR (n = 14) and Group 2-WR (n = 14): The specimens in these groups were immersed in whitening oral rinse (Listerine Advanced White mouth rinse) for 1 min twice daily for 14 consecutive days in a humid environment at 37 °C.

Group 1-CP (n = 14) and Group 2-CP (n = 14): 22% carbamide peroxide advanced tooth whitening system (Pola Night) was used for the specimens in these groups. A standardized volume of the whitening gel was applied from the syringe to the attachment reservoirs of the attachment templates and pressed against the attachments on the tooth surfaces. The epoxy resin master models were kept in a dark environment for 45 min, and after treatment, the templates were taken out.

**Fig. 1:**
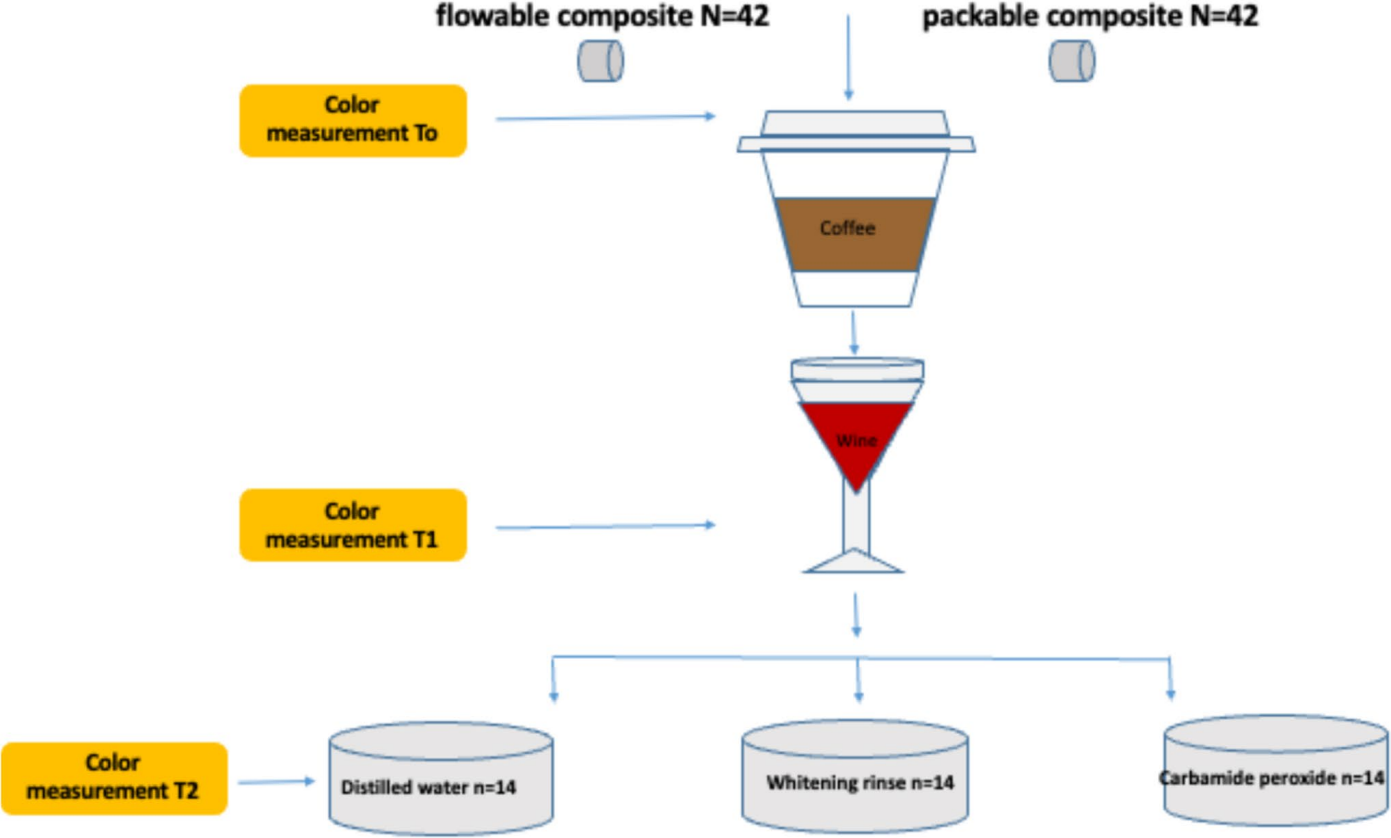
Experimental flowchart and groups

Since it is recommended that all whitening systems require two weeks of this regimen for maximum effect, we conducted a two-week whitening process for both groups. The whitening cycle for both the oral rinse and carbamide peroxide groups was conducted for 14 consecutive days. The experimental flowchart and groups are schematized in Fig. 1.

At the end of each whitening procedure, the specimens were thoroughly rinsed with distilled water for one minute and then dried. The specimens were then immersed in distilled water at 37 °C to serve as a storage media between whitening procedures. The distilled water was renewed daily.

### Color measurements

After the whitening processes, color (T2) measurements of all attachment samples (N = 84) were repeated by the same calibrated author of the study (E.A.) in the same room under the same lighting conditions, at the same time of the day. Color measurements were conducted in a color measurement cabin to ensure standardization. The light power in the color cabin was set to 6500 K in accordance with CIE standards (Master TL-D 90 Graphica 18W/965, Philips, Signify, Poland). The inside of the color measurement cabin was covered with a gray floor, and the measurements were made on a white background.

Color measurements were made from the center of the attachments with a digital spectrophotometer (VITA Easyshade V; VITA Zahnfabrik, Bad Säckingen, Germany) after calibration of the device in accordance with the manufacturer’s instructions. The optic part of the device was held perpendicular to the attachment’s labial surface. Color measurement on each model was made from the right second molar to the left second molar tooth. Specially prepared non-transparent white papers including holes suitable for the attachment dimensions were used to prevent the spectrophotometer from being affected by the color of gray epoxy resin models. Three measurements were taken from the central areas, and the mean values for the L* (lightness from white to black), a* (red or green), and b* (yellow or blue) parameters were applied into the CIEDE2000 formula [44]. Before each measurement, the device was calibrated in accordance with the company’s recommendation. *ΔE*_*00*_ formula was used to determine color differences between T0-T1 (*ΔE*_*001*_*)*, T1-T2 (*ΔE*_*002*_*)* and T0-T2 (*ΔE*_*003*_*)* color measurement periods as following:

$${\Delta \text{E}}_{00}=\sqrt{\left[{\left(\frac{\Delta L{\prime}}{{K}_{L}{S}_{L}}\right)}^{2}+{\left(\frac{\Delta C{\prime}}{{K}_{C}{S}_{C}}\right)}^{2}+{\left(\frac{\Delta H{\prime}}{{K}_{H}{S}_{H}}\right)}^{2}+{R}_{T}\left(\frac{\Delta C{\prime}}{{K}_{C}{S}_{C}}\right)\left(\frac{\Delta H{\prime}}{{K}_{H}{S}_{H}}\right)\right]}$$

The formula uses the differences of three CIELAB metrics: ΔL* (lightness), ΔC* (chroma), and ΔH* (hue). The RT function was added to provide a better performance in the equation, adjusting chromatic differences in the blue region. The formula also considers the weighting functions for lightness (SL), chroma (SC) and hue (SH), and their parametric factors (KL, KC, KH). Finally color changes were analyzed on the basis of an acceptability threshold of 50:50% (AT: ΔE_00_ = 1.8) and a perceptability of 50:50% (PT:ΔE_00_ = 0.8) taking into account the recommendation by the ınternational Organization for Standardization [44–46].

For the attachments’ whiteness change assessment before and after bleaching procedures, Whiteness Index for Dentistry (WI_D_) formula was performed as following:

$${\text{WI}}_{\text{D}}={0.511L}^{*}-{2.324a}^{*}-{1.100b}^{*}$$

Considering the formula the followings were calculated:

$${\Delta \text{WI}}_{\text{D}1}={\text{WI}}_{\text{D}}\left(\text{T}2\right)-{\text{WI}}_{\text{D}}\left(\text{T}0\right)$$

$${\Delta \text{WI}}_{\text{D}2}={\text{WI}}_{\text{D}}\left(\text{T}2\right)-{\text{WI}}_{\text{D}}\left(\text{T}1\right)$$

For the analysis, the 50:50% whiteness perceptibility threshold of 0.72 units (WPT = 0.72) and the 50:50% whiteness acceptability threshold of 2.60 units (WAT = 2.60) were accepted [47].

#### Statistical analysis

Descriptive statistics of the data were given as mean, standard deviation, median, minimum, and maximum. The assumption of normal distribution was verified using the Shapiro–Wilk test, and variance homogeneity was assessed with the Levene test. In cases where the normality assumption was met, the Independent Samples T-test was used to compare two independent groups, and in cases where the assumption was not met, the Mann–Whitney U test was used. The ANOVA test was used to compare three or more independent groups with normal distribution, and the Kruskal–Wallis test was used when there was no normal distribution. The Two-Way ANOVA test was conducted to examine the differences among three independent groups, assuming normality and including the interaction effect. Post Hoc Bonferroni and corrected Bonferroni tests were used to identify the group or groups that generated the difference. The analyses were performed using IBM SPSS 25 program. P < 0.05 was considered as statistically significant.

## Results

Regardless of the whitening agent groups, the ΔE_001_ values after staining for flowable and packable composites are presented in Table 2. Regarding the color difference measurements, ΔE_001_ values for the packable composite group (Group 2) were significantly higher than those of the flowable composite group (Group 1) (p < 0.001).

**Table 2:** Comparison of the *ΔE*_*001,*_* ΔE*_*002,*_* ΔE*_*003,*_ ΔWI_D1,_ and ΔWI_D2_ values of the tested groups

Variables		Flowable (Group 1) (N = 42)	Packable (Group 2) (N = 42)	
		Mean ± SD	Mean ± SD	Test statistics
ΔE_001_		6.32 ± 3.52^A^	14.94 ± 4.01^B^	−10.473
ΔE_002_	DW	6.84 ± 4.14^A^	4.7 ± 2.58^A^	1.646
	WR	5.12 ± 2.82^A^	6.29 ± 5.57^A^	−0.230†
	CP	6.95 ± 3.04^A^	10.69 ± 6.09^A^	−2.053
	Test statistics	2.626‡	7.621‡	
	p-between whitening agents	0.269	0.022* (2 DW-2 CP;p = 0.023)	
ΔE_003_	DW	5.52 ± 3.49^A^	14.99 ± 2.21^B^	−4.181†
	WR	5.46 ± 2.53^A^	12.4 ± 2.64^B^	−7.108
	CP	4.8 ± 1.78^A^	8.29 ± 2.79^B^	6.993
	Test statistics	0.380‡	24.482	
	p-between whitening agents	0.827	< 0.001* (2 DW-2 WR;p = 0.032) (2 DW-2 CP;p < 0.001) (2 WR-2 CP; p < 0.001)	
ΔWI_D1_	DW	−5.74 ± 8.73^A^	−27.06 ± 6.28^B^	−4.043†
	WR	−6.5 ± 8.45^A^	−23.4 ± 5.84^B^	−4.043†
	CP	−1.5 ± 4.81^A^	−15.29 ± 5.59^B^	−2.554
	Test statistics	1.781	19.071‡	
	p-between whitening agents	0.182	< 0.001* (2 DW-2 CP; p < 0.001) (2 WR-2 CP; p = 0.024)	
ΔWI_D2_	DW	−1.39 ± 7.24^A^	1.92 ± 4.09^A^	−1.489
	WR	4.03 ± 4.28^A^	5.05 ± 9.72^A^	−0.735†
	CP	7.09 ± 5.22^A^	21.01 ± 19.71^B^	−2.849†
	Test statistics	7.907	19.052‡	
	p-between whitening agents	0.001* (1 DW-1 WR; p = 0.049) (1 DW-1 CP; p = 0.001)	< 0.001* (2 DW-2 CP; p < 0.001) (2 WR-2 CP; p = 0.024)	

*p < 0.05 statistically significant; †: Mann Whitney U test; ‡: Kruskal Wallis test

DW: Distilled water; WR:Whitening Rinse; CP: Carbamide Peroxide; SD: Standard Deviation.

*ΔE*_*001*_ = T1-T0; *ΔE*_*002*_ = T2-T1; and *ΔE*_*003*_ = T2-T0.

ΔWI_D1_ = WI_D_ (T2) − WI_D_(T0).

ΔWI_D2_ = WI_D_ (T2) − WI_D_ (T1).

Note: Different uppercase letters (A, B) in the same row indicate statistically significant differences between Group 1 and Group 2 regarding whitening agents.

In the flowable composite group (Group 1), no significant differences were observed among the various whitening agents (DW, WR, and CP) regarding ΔE_002_, ΔE_003_, and ΔWI_D1_ measurements. On the other hand, the ΔWI_D2_ measurement in Group 1 showed statistically significant differences among the DW, WR, and CP groups (p = 0.001). The measurements in the WR and CP subgroups of Group 1 were significantly higher than those in the DW group (p = 0.049 and p = 0.001). (Table 2).

In the packable composite group (Group 2), the ΔE_002_ value for subgroup CP was significantly higher than that for the DW group (p = 0.023). For the ΔE003 measurement in Group 2, statistically significant differences were found among the DW, WR, and CP groups (p < 0.001). In Group 2, the ΔE_003_ measurement for the DW group was higher than the measurements for the CP group (p < 0.001) and the WR group (p = 0.032). Additionally, the ΔE_003_ measurement for the WR group was greater than that of the CP group (p < 0.001). According to the ΔWI_D1_ measurement in Group 2, statistically significant differences were found between the subgroups (p < 0.001). The ΔWI_D1_ measurement of the CP group was lower than that of the DW (p < 0.001) and WR (p = 0.014) groups. According to the ΔWID2 measurement in Group 2, CP measurements were significantly higher than those of DW (p < 0.001) and WR (p = 0.024) measurements. (Table 2).

**Fig. 2:**
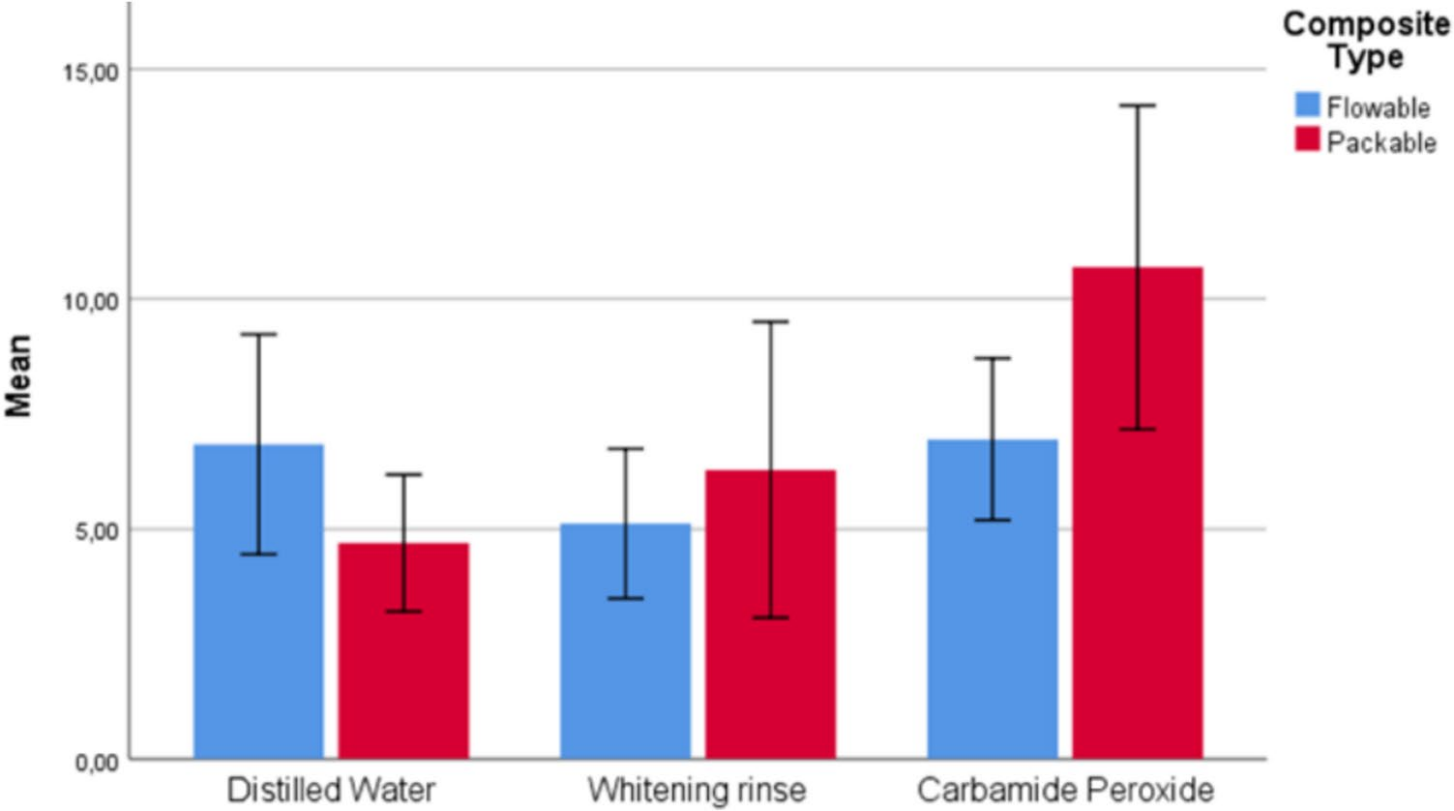
Box plot of the distribution of ΔE_002_ measurements according to composite and whitening agent groups

**Fig. 3:**
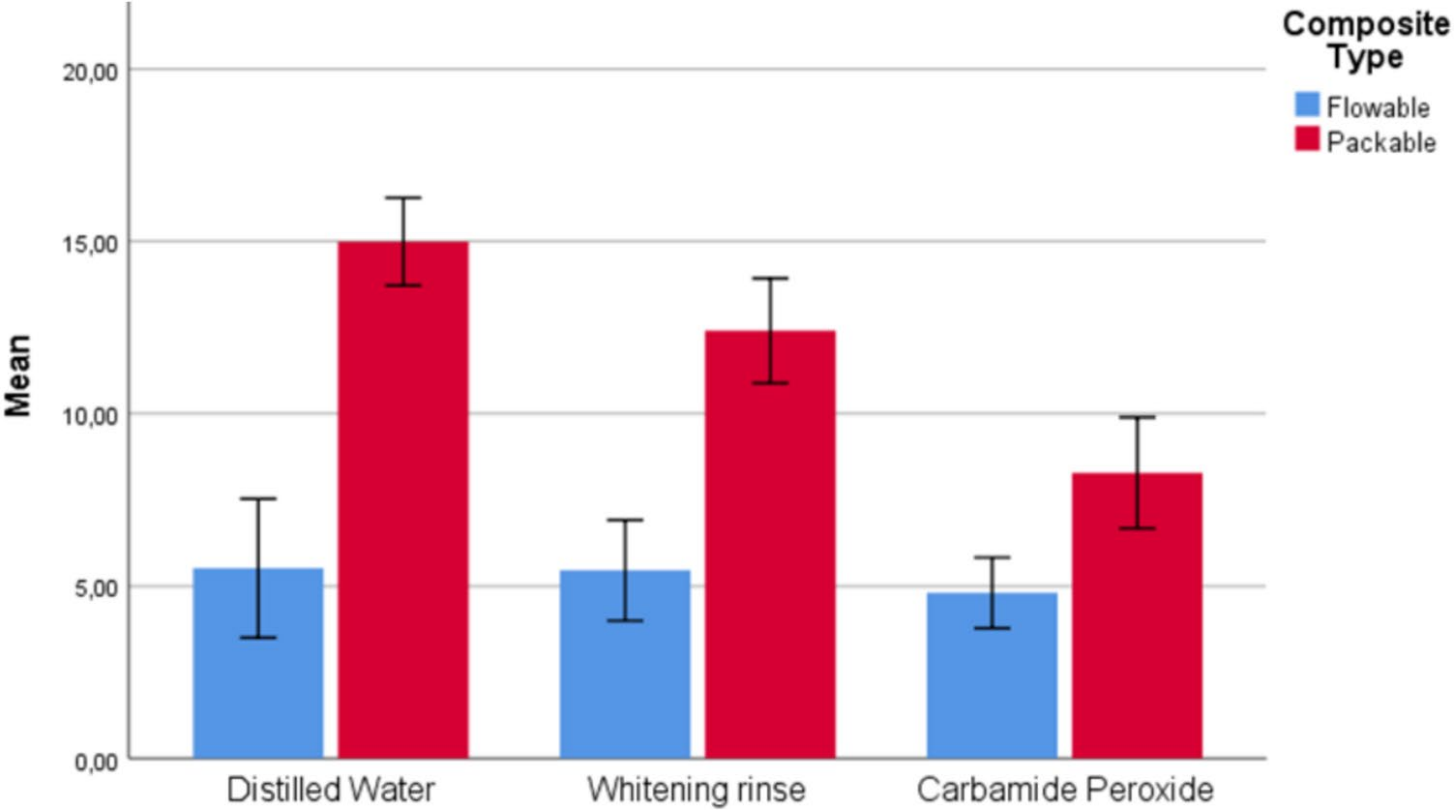
Box plot of the distribution of ΔE_003_ measurements according to composite and whitening agent groups

**Fig. 4:**
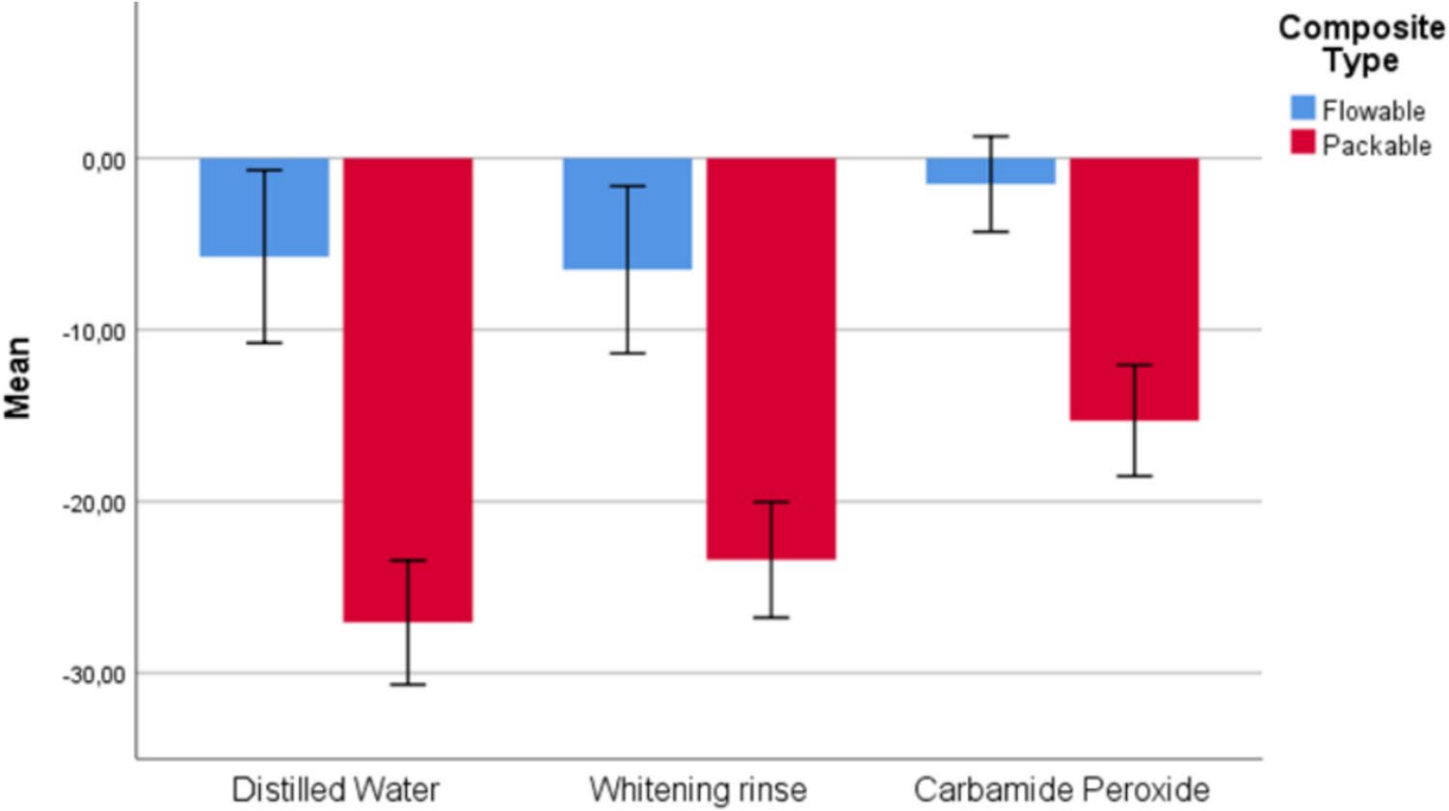
Box plot of the distribution of ΔWI_D1_ measurements according to composite and whitening agent groups

**Fig. 5:**
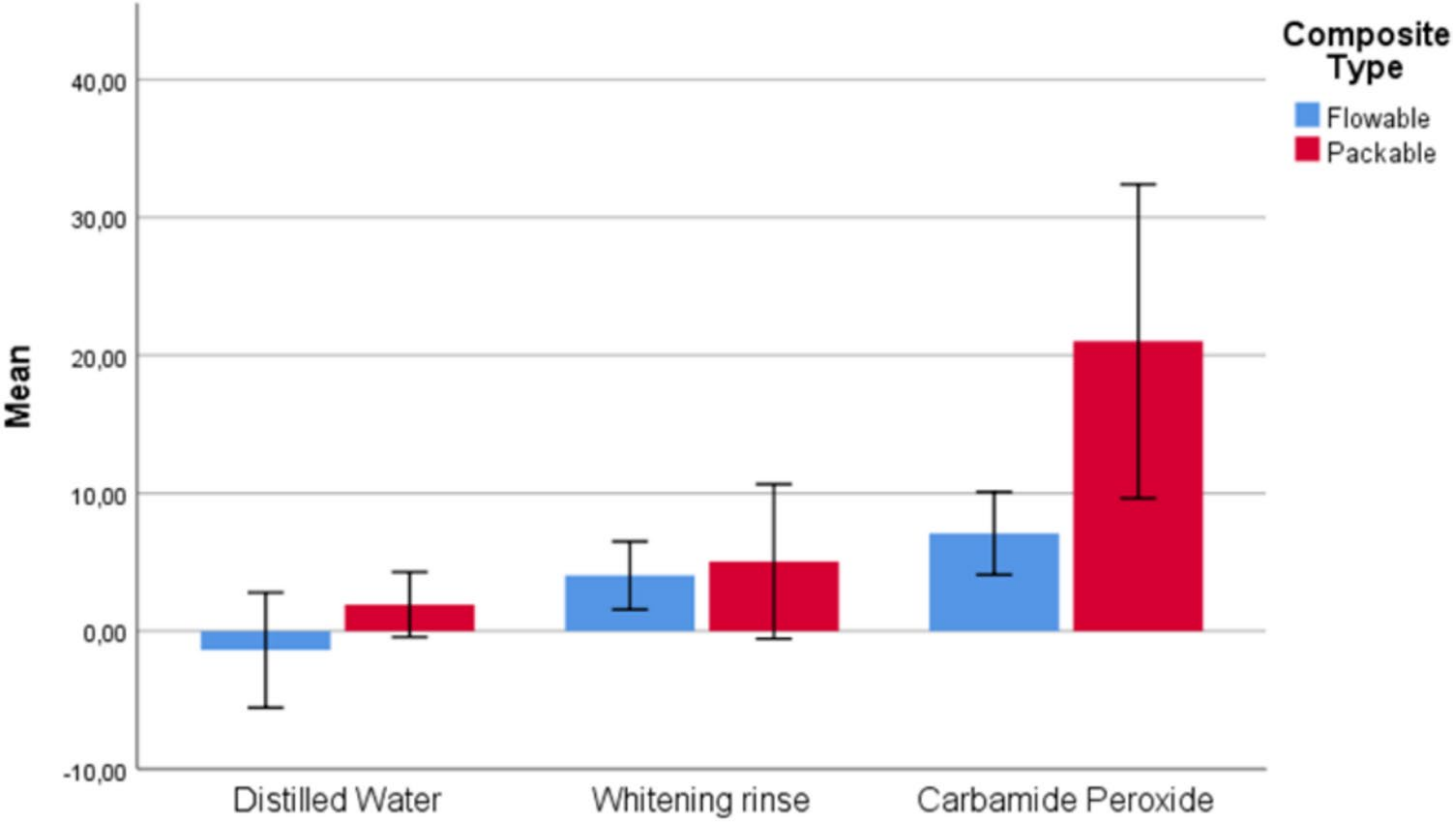
Box plot of the distribution of ΔWI_D2_ measurements according to composite and whitening agent groups

The results of comparing flowable and packable composite groups (1 and 2), considering different whitening agents, are shown in Table 2 and Figs. 2, 3, 4, and 5. As a result of the analyses conducted on whitening types, statistically significant differences were found between Group 1 and Group 2 for DW, WR, and CP whitening types concerning ΔE_003_ (p < 0.001) and ΔWI_D1_ (p < 0.05) measurements, as well as between the composite groups for the CP whitening type regarding ΔWI_D2_ (p = 0.004) measurements. For all significant differences, measurements for the packable composite group (Group 2) were found to be greater than those for the flowable composite group (Group 1).

## Discussion

Considering the higher duration of orthodontic treatment, it is important to maintain or enhance the smile aesthetics taking into account the patient’s esthetic expectations. Tooth whitening can also be used during orthodontic treatment to meet this demand by offering social and psychological benefits. [48, 49]. During clear aligner treatments, composite attachments customized in different shapes and sizes are often necessary to enhance complex tooth movements [50]. Sword and Haywood emphasized the effectiveness of home dental bleaching during clear aligner treatment, showing positive results in a case report study [51]. They also showed bleaching on the tooth areas under the composite attachments. In a recent study conducted by Kaji et al. [52], it was concluded that the attachments did not impede tooth whitening on the tooth surface beneath them.

From a clinical perspective, aesthetic dissatisfaction can also arise during clear aligner treatment due to the external pigmentation of composite attachments. Therefore, increased whitening of dental composites, besides tooth whitening, would be desirable for both clinicians and patients. For this reason, the study aimed to evaluate the effects of carbamide peroxide and whitening rinse solutions, both used for at-home whitening protocols, on the color properties of flowable nanohybrid composite and packable hybrid composite aligner attachments. The duration of the whitening process for this in-vitro study was selected as 14 days, as it is the most commonly used period [53].

For aligner attachment production, while flowable composites offer superior handling properties [4], packable composites deliver greater resistance to deformation and enhanced mechanical performance [5]. Consequently, two different composites with varying physical properties and filler volume percentages from the same manufacturer were chosen, both possessing the A2 variant shade property.

The current results indicated that both the flowable and packable composite aligner attachments, which were immersed in coffee and red wine solutions, displayed ΔE_001_ values that were unacceptable and imperceptible. One reason for this result may be due to the fact that the pigmentation ability of resin composites relates to their hydrophilic properties. Due to the sorption and hydrolysis properties of the monomers in the resin composites, they can absorb not only water but also water-soluble coloring agents [54, 55]. Another reason for unacceptable ΔE_001_ values after staining for both flowable and packable composite attachments could be unpolished attachment surfaces. This is because the resin attachments do not undergo the traditional polishing stage as specified by the manufacturer, since they support tooth movements [19]. In support of this reason, Duc et al. demonstrated a 30% greater color change in unpolished resin surfaces compared to polished ones [18]. Similar to the findings of the present study, Chami et al. [33] presented visually unacceptable color changes of different resin composites (Filtek Z350 XT, Filtek Z250 XT, Z100 resin composites, and Transbond XT orthodontic resin) used for orthodontic clear aligner attachments when immersed in red wine and coffee.

When comparing two main composite attachment groups, the results of this study indicated that G-ænial posterior composite resin exhibited significantly higher ΔE_001_ values than G-ænial Universal flowable composite resin after staining with coffee and red wine solutions. This result indicated that universal flowable resin was more resistant to coffee and wine staining than packable posterior resin type.

Erçin et al. [34] compared single-shade Omnichroma resin, flowable bulk-fill Tetric PowerFlow resin, and GC Aligner Connect, which was produced as a build-for-attachment composite resin, with regard to color changes using an intraoral surface scanner. According to their findings, Tetric PowerFlow flowable bulk-fill resin was more susceptible to coffee staining than GC Aligner Connect and Omnichroma resins. In the study by Özsoy et al. [35], G-aenial Universal Flo was compared to Filtek Ultimate packable composite on human premolars with respect to color changes of two different designs of aligner attachments after external discoloration. Similar to our results, the flowable composite group showed less coloration than the packable group for both attachment designs. They recommended a flowable nanocomposite resin for the anterior region, where aesthetics is an important factor, as we also advise based on our findings results. G-aenial Universal Flo features uniformly dispersed, ultra-fine silane-treated filler particles [37], which enhance the gloss of unpolished surfaces over time thanks to their self-polishing properties. The production of the resin with a new silane treatment improved the hydrolytic stability and durability of the adhesive [56]. Therefore, these enhanced properties may lead to greater resistance to staining in the flowable nano-hybrid composite attachment group compared to the hybrid packable composite group in the present study. Yildiz et al. [2] evaluated the discoloration resistance of composite attachments with varying physical properties in a simulated environment, including thermal cycles and the insertion and removal of aligners. After the 24th week of experimental periods, similar to our findings, G-Aenial Universal Injectable was less discolored than the other samples (Aligner Connect and Tetric Prime and Tetric Evoflow combination).

In the present study, after staining all specimens, whitening agents were applied to simulate chemical bleaching and whitening processes using carbamide peroxide and a whitening rinse solution, respectively. The null hypothesis of the current in-vitro study was rejected for the G-aenial posterior packable composite group (Group 2) because the value of ΔE_002_ for subgroup CP was significantly higher than that of the DW group (p = 0.023). Additionally, the ΔE_003_ measurement for the WR group was greater than that of the CP group (p < 0.001). All these results indicated that, especially in the packable composite group, carbamide peroxide whitening agent significantly affected the color of the attachments compared to the control distilled water group and whitening rinse solution group. On the other hand, for the flowable composite group, the null hypothesis was accepted and three different subgroups (distilled water, whitening rinse, and carbamide peroxide) did not differ among each other regarding ΔE_002_ and ΔE_003_ measurements.

In the study of Ghaemi et al. [29], a micro-hybrid (Filtek Z250, 3 M ESPE, St. Paul, MN, USA), a nanohybrid (Filtek Z‑350XT, 3 M‑ESPE), and a nano-filled composite (Filtek Z‑250XT, 3 M‑ESPE) resins were compared according to the effects of bleaching and staining solutions on stainability and color stability. A 15% carbamide peroxide for seven hours a day for a total of 14 days was used as a bleaching agent. Different from our study design, they exposed the composites to three different staining solutions for two weeks following the bleaching protocol. In the end, they found that carbamide peroxide did not significantly affect the color of composite specimens. Also, the stainability of composites did not increase after bleaching. In our study, however, we first applied staining and then bleaching. Unlike the findings of this study, the carbamide peroxide agent positively contributed to a significant color change, particularly in the hybrid composite resin group (Group 2:G-ænial Posterior packable).

In a recent study, [26] microhybrid and nanohybrid composites were whitened with hydrogen or carbamide peroxide in a simulated procedure, and were evaluated regarding color changes immediately after whitening to allow comparison of the effects of whitening agents. The color changes of both composite types reached a 50:50% perceptibility, but remained below the 50:50% acceptability threshold. No significant difference in color changes between the tested composites for either whitening protocol was attributed to the similarities in composition (Herculite XRV and Herculite XRV Ultra from Kerr company) of the composites in terms of resin matrix. Also, in our study, nano-hybrid flowable and microfilled hybrid packable composites from the same manufacturer did not show statistically significant differences between the staining and bleaching protocols regarding ΔE_002_ measurement, despite having different filler types, sizes, and distributions. On the other hand, unlike the findings of the study by Savic-Stankovic et al. [26], which reported similar effects on the composite color of the tested 16% carbamide peroxide and 40% hydrogen peroxide whitening agents, we observed statistically significant differences in the packable hybrid composite group between carbamide peroxide and whitening rinse solutions, particularly concerning the ΔE_003_ measurement from baseline to the post-bleaching periods. This result could be attributed to the fact that carbamide peroxide showed a statistically significant color change effect on packable hybrid composite attachments when compared to the control distilled water group between the staining and bleaching periods.

The Whiteness Index for Dentistry (WID) was calculated to assess the changes in attachment whiteness after using different whitening agents. According to this index, higher value of WI_D_ indicates higher whiteness values [57]. There was a decreasing pattern for WI_D1_ from baseline to after staining and whitening protocols. This result could be attributed to the fact that higher values were observed at the beginning due to the absence of treatment, and specimens became darker as a result of 14 days of staining, even though they underwent a whitening protocol. Therefore negative values were observed for WI_D1_ values from baseline to the final measurement. On the other hand, after the whitening protocols, in both flowable (Group 1) and packable composite attachment groups (Group 2), there was an increase in the WI_D2_ values for both whitening rinse and carbamide peroxide subgroups. While in Group 1, both whitening rinse and carbamide peroxide whitening agents showed significantly higher WI_D_ changes after staining compared to the control distilled water groups, in Group 2, carbamide peroxide agent showed higher value changes compared to both the control and the whitening rinse subgroups. This result emphasizes that both carbamide peroxide and whitening rinse solution have a significant effect on attachment whitening in flowable composite types, while carbamide peroxide causes a more effective increase in the whitening index compared to the whitening rinse in the packable composite group. For ΔWI_D2_, the value obtained for the packable composite in the DW group without bleaching showed that the samples' whiteness increased, while for the flowable composite group in the DW subgroup it was decreased***.*** This result may be due to the significantly higher coloration in the packable composite group, as indicated by the ΔE_001_ value. The excessive coloration in this group may have led to a decrease in coloration when placed in distilled water, resulting in an increase in whiteness index. According to the results of the present study, it can be suggested that even if 6 days of staining promoted a severe color change on aligner attachments, this change did not impair the ability of the tested whitening agents to promote attachment whitening. Erturk-Avunduk [28] compared a nanohybrid resin composite with nine different bulk-fill resin composites that were treated with hydrogen peroxide and carbamide peroxide after being stained with tea, coffee, and red wine. In contrast to the findings of our present study, for all the resins and staining beverages, no significant difference in ΔWI_D1_ and ΔWI_D2_ values was detected between the bleaching agents indicating that the type of bleaching did not affect the whiteness of tested composites. In our study, in the hybrid packable composite attachment group, carbamide peroxide was more effective on the whiteness compared to the whitening rinse solution, and the differences between the studies may arise from the different whitening agents preferred in the methods.

Another category of mouthwashes introduced to the dental market is the bleaching group, and the effects of peroxide ingredients in these mouthwashes on the resinous matrix are questionable. Hamdy et al. [32] investigated the color changes of the nanohybrid composite after being treated with three different types of mouthwashes (Chlorhexidine-based mouthwash, Listerine Green Tea mouthwash, and Colgate Optic White Whitening Mouthwash). According to their findings, immersion in a bleaching mouthwash simulating two years of clinical use resulted in a positive ΔL value, indicating that the resin composite became lighter. In line with the findings of Hamdy et al. [32], we also observed that the whitening rinse mouthwash was effective in the stained nano-hybrid flowable attachment group, showing a significant change in WI_D2_ compared to the control distilled water group. This result could be considered an important clinical finding for both patients and clinicians, as whitening rinse is an easily accessible product. The effect of Listerine whitening mouthwash on color recovery of discolored composite resins was also studied in another in-vitro experimental study [31]. IPS Empress Direct and Filtek Z350XT universal composite resins were immersed in the coffee solution for 7 days, and thereafter in Listerine (Johnson & Johnson Consumer Inc., Skillman, NJ, USA) for 4 min daily for a total of 56 days at room temperature. After immersion in mouthwash, color recovery was significantly greater in Z350XT, and the color recovery was also statistically significant in each group. Different from our study design, the immersion in mouthwash was longer; therefore, it can be thought that it did not provide as effective whitening as carbamide peroxide in our study.

In a recent study [58], it was mentioned with the results that short-term regular use of whitening mouth rinses (Crest 3D White and Listerine Advanced White) can recover color and increase the perception of whiteness without significantly increasing the roughness and hardness of resin composites. Similarly to our results, an at-home whitening agent caused a significantly greater color change than whitening mouthwash on micro-hybrid and nanohybrid resin composite in the study by Vural et al. [59]. They emphasized that WMR contains only 2% HP, and the whitening mechanism relies on the removal of extrinsic stains by silica.

It should also be noted that the whitening process may affect the biomechanical properties of aligners, which could also indirectly affect the clinical effectiveness. In a recent randomized controlled study conducted by Jin et al. [60], marked tooth color changes were achieved with vacuum-formed retainers (polyurethane based) combined with %10 carbamide peroxide, on the other hand, a decrease in tensile strength and an increase in hardness and internal roughness was observed for retainers. Although clear aligners are changed every two weeks, considering that the application of carbamide peroxide for approximately two weeks may cause a change in the mechanical properties of the plates, perhaps long-term use may be preferred when switching to the next aligner, or a vacuum-formed plate may be printed and whitening process could be carried out with it instead of the aligner plate.

Another point is that we did not measure the surface roughness of the attachments, a factor that would be interesting to investigate further in future studies. Although the whitening agents created positive changes in the colors of the attachments, negative changes in surface roughness would be created. They may also cause plaque accumulation and indirect color changes on attachment surfaces.

The current study has certain limitations. One major limitation is the lack of simulating an oral environment. Additionally, another limitation is not assessing the daily brushing impact on the attachment surfaces. The usual brushing method, along with the continuous washing effect of saliva, would diminish the staining effect. Therefore, further studies could be handled by simulating the aging procedure, thermal alterations, and the presence of saliva with in-vivo study designs. Besides, further studies are required to evaluate the effects of daily tooth brushing techniques combined with whitening agents on the discoloration of composite aligner attachments.

## Conclusions

- The color change of the packable micro-filled hybrid composite attachment was more pronounced than that of the flowable nanohybrid composite attachment after immersion in coffee and red wine. The color changes for both groups were above the acceptability and perceptibility thresholds.
- Between the bleaching and staining stages, particularly in the packable composite group, the carbamide peroxide whitening agent had a significant impact on the color and whiteness of the attachments compared to the control distilled water group.
- The color and whiteness changes of the attachments in the packable composite group were significantly greater than those in the flowable composite group, considering different whitening factors agents.

## Data Availability

No datasets were generated or analysed during the current study.
